# Comprehensive Analysis of Ozanimod-Related Adverse Events Based on the FAERS Database

**DOI:** 10.14740/wjon2777

**Published:** 2026-06-25

**Authors:** Zi Ru Zhou, Qun Yan Zhou, Yue Shen, Xiao Yi Zhao, Qiang Zhan, Jing Sun, Zhong Xia Chen

**Affiliations:** a Department of Gastroenterology, The Affiliated Wuxi People’s Hospital of Nanjing Medical University, Wuxi, Jiangsu, China; b These authors contributed equally to this study.

**Keywords:** Ozanimod, Adverse event, FAERS, Ulcerative colitis, Safety

## Abstract

**Background:**

Ozanimod, a selective sphingosine-1-phosphate receptor modulator, has been approved for the treatment of moderately to severely active ulcerative colitis (UC) and relapsing forms of multiple sclerosis (MS). However, its postmarketing adverse event (AE) reporting profile requires further characterization.

**Methods:**

AE reports involving ozanimod from Q2 2020 to Q1 2025 were retrieved from the FDA Adverse Event Reporting System (FAERS) database. Frequency-based and Bayesian disproportionality methods were applied, including the reporting odds ratio (ROR), proportional reporting ratio (PRR), Bayesian confidence propagation neural network (BCPNN), and empirical Bayesian geometric mean (EBGM), to identify pharmacovigilance reporting signals.

**Results:**

A total of 15,910 AE reports involving ozanimod were retrieved, of which 7,305 reports listed ozanimod as the primary suspected drug. Most reports involved patients aged 18–64 years (67.5%), and more reports were submitted for female patients than for male patients. Frequently reported events included fatigue, headache, dizziness, and back pain. Labeled or clinically recognized safety events, such as decreased lymphocyte count, decreased heart rate, hypertension, macular edema, and liver test abnormalities, were also detected as reporting signals. Disease-related Preferred Terms, including MS relapse, MS, UC, and proctitis ulcerative, showed strong disproportionality signals but may reflect underlying disease activity, treatment failure, indication, or reporting context rather than toxic adverse drug reactions. In addition, several hypothesis-generating FAERS signals not explicitly listed in current product labeling were observed, including depression, feeling of despair, decreased interest, penile swelling, and papillary thyroid cancer. These findings require cautious interpretation because FAERS cannot establish incidence, absolute risk, or causality.

**Conclusions:**

This study provides a postmarketing pharmacovigilance signal profile of AE reports involving ozanimod. The findings support continued monitoring of labeled safety concerns, careful interpretation of disease-activity-related reports, and further validation of selected unlabeled signals in prospective studies or better-controlled real-world datasets.

## Introduction

Ulcerative colitis (UC), one of the two major forms of inflammatory bowel disease (IBD), is a chronic inflammatory condition of the colon [1]. UC has a relapsing-remitting course, which necessitates different therapeutic approaches to induce and maintain remission. There is no known cure for UC, and thus, the goals of treatment are resolving symptoms, improving quality of life, and preventing and treating complications [2]. Studies have demonstrated that within 5 years of diagnosis, approximately 20% of those with UC are hospitalized, and 7% require colectomy [3–6]. Understanding the pathogenesis of UC has led to the development of different classes of biologics and oral small molecules that have expanded treatment options for UC [7]. Nearly 20 years ago, the advent of antitumor necrosis factor biologics revolutionized therapeutics for UC, enabling better disease control in terms of increasing the rates of mucosal healing, deep remission, and corticosteroid-free remission and improving quality of life. Biologics with other targets were later approved for the treatment of moderate-to-severe UC. However, treatment with biologics has several limitations, including limited efficacy, primary nonresponse, secondary loss of response, immunogenicity, and parenteral administration [8].

Drug development has shifted in the past decade toward oral small-molecule therapies, which offer practical advantages such as oral administration, lack of immunogenicity, and relatively short half-lives compared with biologics. Tofacitinib, a Janus kinase (JAK) inhibitor, was the first next-generation oral small-molecule drug approved by the US Food and Drug Administration (FDA) for the treatment of patients with moderate-to-severe UC and remains an important therapeutic option in clinical practice [9]. Its introduction marked a major transition in UC treatment from injectable biologics to oral targeted therapies. However, different classes of small molecules have distinct mechanisms of action, efficacy profiles, and safety considerations. Therefore, the development and evaluation of newer oral agents with alternative and more selective immunomodulatory mechanisms, such as sphingosine-1-phosphate (S1P) receptor modulators, are clinically important for expanding therapeutic options and optimizing individualized treatment strategies [10].

S1P is a bioactive lysophospholipid that regulates immune-cell trafficking through five G-protein-coupled receptor subtypes, S1PR1–S1PR5. Among these receptors, S1PR1 is highly expressed on lymphocytes and is essential for sensing the S1P concentration gradient between lymphoid tissues and the circulation, thereby promoting lymphocyte egress from lymph nodes into peripheral blood and subsequently to inflamed tissues [11, 12]. In IBD, inhibition of lymphocyte trafficking represents a rational therapeutic strategy because excessive recruitment of activated lymphocytes to the intestinal mucosa contributes to persistent inflammation [13]. S1PR modulators have a seemingly paradoxical mechanism of action. Although they initially act as receptor agonists by binding to S1PRs, sustained receptor engagement induces S1PR1 internalization, degradation, or desensitization on lymphocytes. As a result, lymphocytes become unable to respond to the S1P gradient and are retained within lymphoid tissues, leading to a reversible reduction in circulating lymphocytes and decreased migration of pathogenic lymphocytes into the inflamed intestinal mucosa. Therefore, S1PR modulators are often described pharmacologically as “functional antagonists” of S1PR1 despite their initial agonistic binding activity [11–13]. Compared with the first-generation S1PR modulator fingolimod, which interacts with multiple S1PR subtypes including S1PR1, S1PR3, S1PR4, and S1PR5, newer S1PR modulators have been developed to improve receptor selectivity and potentially reduce off-target adverse effects. Ozanimod is a selective oral S1PR modulator with high affinity for S1PR1 and S1PR5 and minimal activity at S1PR2, S1PR3, and S1PR4 [11, 14]. This receptor-binding profile is clinically relevant because S1PR1 modulation is primarily responsible for lymphocyte sequestration, whereas avoidance of S1PR3 may theoretically reduce cardiovascular and pulmonary off-target effects associated with less selective S1PR modulation [12, 13]. Other newer agents also differ in receptor selectivity: siponimod mainly targets S1PR1 and S1PR5, ponesimod is relatively selective for S1PR1, whereas etrasimod targets S1PR1, S1PR4, and S1PR5. These differences in receptor binding, pharmacokinetics, and reversibility may contribute to differences in lymphocyte recovery, dosing requirements, efficacy profiles, and adverse-event patterns among S1PR modulators [13, 15].

Oral ozanimod (brand name Zeposia^®^) was originally approved for relapsing forms of multiple sclerosis (MS). On May 27, 2021, the FDA approved ozanimod 0.92 mg for the treatment of adults with moderately to severely active UC, making it the first oral S1P receptor modulator approved for this indication. In November 2021, the European Commission also approved ozanimod for adult patients with moderately to severely active UC who had an inadequate response, loss of response, or intolerance to conventional therapy or biologic therapy [12, 15]. Despite its receptor selectivity and oral convenience, ozanimod is associated with clinically relevant safety concerns, including infections related to lymphocyte reduction, bradyarrhythmia or atrioventricular conduction delay during treatment initiation, liver enzyme elevation, macular edema, hypertension, and rare opportunistic infections such as progressive multifocal leukoencephalopathy [12, 15]. The potential adverse reactions and associated reporting signal of ozanimod in large-scale postmarketing use urgently require comprehensive evaluation. The FDA Adverse Event Reporting System (FAERS) is a widely used pharmacovigilance database that compiles reports of adverse events (AEs) related to drug use globally, providing valuable clinical data for drug safety monitoring [16–22]. This study aims to mine and analyze the AEs of ozanimod via the FAERS database to understand its safety profile in the real world.

## Materials and Methods

### Data sources

The data for this study were obtained from the FAERS database, which includes all the AE reports from the second quarter of 2020 to the first quarter of 2025. The dataset encompasses several categories of records: demographic information (DEMO), drug utilization information (DRUG), treatment outcome information (OUTC), AE information (REAC), report source information (RPSR), and treatment duration information (THER). Because FAERS is a spontaneous reporting database, disproportionality analyses can identify reporting signals but cannot establish incidence, absolute risk, or causal relationships. Therefore, all AE signals identified in this study should be interpreted as hypothesis-generating pharmacovigilance findings rather than confirmed drug-related adverse reactions.

### Data processing

Ozanimod was identified in the DRUG table using both the generic name and brand name. Drug-name cleaning was performed before case selection. Specifically, all drug names were converted to uppercase, and spaces, punctuation marks, special characters, and common spelling variations were standardized. Records containing “OZANIMOD” or “ZEPOSIA” were manually reviewed and harmonized as ozanimod to ensure comprehensive retrieval.

Only reports in which ozanimod was recorded as the primary suspect drug (ROLE_COD = “PS”) were included in the ozanimod-exposed group. Reports in which ozanimod appeared only as a secondary suspect drug, concomitant drug, or interacting drug were excluded from the primary exposure definition. The comparator group consisted of all other deduplicated FAERS reports during the same study period in which the primary suspect drug was not ozanimod. Thus, the disproportionality analysis compared AE reporting for ozanimod as the primary suspect drug with reporting for all non-ozanimod primary suspect drugs in FAERS during the same time window.

Duplicate reports were removed according to the FDA-recommended procedure. The PRIMARYID, CASEID, and FDA_DT fields were extracted from the DEMO table, and reports were sorted by CASEID, FDA_DT, and PRIMARYID. For reports with the same CASEID, the report with the latest FDA_DT was retained. If multiple reports shared the same CASEID and FDA_DT, the report with the highest PRIMARYID was retained. In addition, reports listed in the FAERS deleted-report files were excluded after deduplication.

AEs were coded according to MedDRA version 27.0 and analyzed at both the System Organ Class (SOC) and Preferred Term (PT) levels [23]. For each PT, a 2 × 2 contingency table was constructed using ozanimod primary-suspect reports as the exposed group and non-ozanimod primary-suspect reports as the comparator group. Concomitant medications were not included in the primary disproportionality calculations and were not used to define exposure. Therefore, the results should be interpreted as reporting signals rather than causal estimates, and residual confounding from concomitant therapies cannot be fully excluded.

### Data analysis

Signal mining was performed via disproportionality analysis methods, primarily frequency-based and Bayesian methods. The frequency-based methods include the reporting odds ratio (ROR) and the proportional reporting ratio (PRR), whereas the Bayesian methods include the Bayesian confidence propagation neural network (BCPNN) and the empirical Bayesian geometric mean (EBGM) [24–27]. The ROR helps minimize bias in events that are reported less frequently, whereas the PRR stands out because of its greater specificity compared with the ROR. The BCPNN excels in integrating and cross-validating multisource data, and the multivariate gamma Poisson shrinker (MGPS) is particularly effective in detecting signals from infrequent events. This study combines the ROR, PRR, BCPNN, and MGPS, leveraging their individual strengths to enhance the detection and validation ranges from different perspectives. This integrated approach allows for more accurate identification of safety signals, reduces false positives through cross-validation, and refines the detection of rare AEs by adjusting thresholds and variance. All four methods are based on proportional imbalance metrics with 2 × 2 tables, as shown in Table 1. To reduce bias introduced by individual algorithms, this study integrates these four methods, with the specific formulas outlined in Table 2.

**Table 1:** Four Grid Table

	Target AEs	Non-target AEs	Total
Ozanimod	*a*	*b*	*a + b*
Non-ozanimod	*c*	*d*	*c + d*
Total	*a + c*	*b + d*	*N = a + b + c + d*

AE: adverse event.

**Table 2:** ROR, PRR, BCPNN, and EBGM Methods, Formulas, and Thresholds

Method	Formula	Threshold
ROR	$\begin{array}{l} \text{ROR}=\frac{a\text{/}c}{b\text{/}d}=\frac{ad}{bc}\\ \text{SE(lnROR)}=\sqrt{\frac{1}{a}+\frac{1}{b}+\frac{1}{c}+\frac{1}{d}}\\ \text{95\% CI}=e^{\text{ln(ROR)}\pm \text{1.96}\sqrt{\frac{1}{a}+\frac{1}{b}+\frac{1}{c}+\frac{1}{d}}} \end{array}$	a ≥ 3 and 95% CI (lower limit) > 1
PRR	$\begin{array}{l} \text{PRR}=\frac{a\text{/}(a+b)}{c\text{/}(c+d)}\\ \text{SE (lnPRR)}=\sqrt{\frac{1}{a}-\frac{1}{a+b}+\frac{1}{c}-\frac{1}{c+d}}\\ \text{95\% CI}=e^{\text{ln(PRR)}\pm \text{1.96}\sqrt{\frac{1}{a}-\frac{1}{a+b}+\frac{1}{c}-\frac{1}{c+d}}} \end{array}$	a ≥ 3 and 95% CI (lower limit) > 1
BCPNN	$\begin{array}{l} \text{IC}={\text{log}}_{\text{2}}\frac{p(x,y)}{p(x)p(y)}={\text{log}}_{\text{2}}\frac{a(a+b+c+d)}{\left(a+b)(a+c\right)}\\ \text{E(IC)}=\log_{2}\frac{\left(a+\gamma 11)(a+b+c+d+\alpha )(a+b+c+d+\beta \right)}{\left(a+b+c+d+\gamma )(a+b+\alpha 1)(a+c+\beta 1\right)}\\ \text{V(IC)}=\frac{\text{1}}{{\text{(ln2)}}^{\text{2}}}\left\{\begin{array}{l} \left[\frac{(a+b+c+d)-a+\gamma -\gamma 11}{\left(a+\gamma 11)(1+a+b+c+d+\gamma \right)}\right]\\ +\left[\frac{\left(a+b+c+d)-(a+b)+\alpha -\alpha 1\right)}{(a+b+\alpha 1)(1+a+b+c+d+\alpha )}\right]\\ +\left[\frac{\left(a+b+c+d)-(a+c)+\beta -\beta 1\right)}{\left(a+c+\beta 1)(1+a+b+c+d+\beta \right)}\right] \end{array}\right\}\\ \gamma =\gamma \text{11}\frac{\left(a+b+c+d+\alpha )(a+b+c+d+\beta \right)}{\left(a+b+\alpha 1)(a+c+\beta 1\right)}\\ IC-2SD=E(IC)-2\sqrt{V(IC)} \end{array}$	IC025 > 0
EBGM	$\begin{array}{l} \text{EBGM}=\frac{a(a+b+c+d)}{\left(a+c)(a+b\right)}\\ \text{95\% CI}=e^{\text{ln(EBGM)}\pm \text{1.96}\sqrt{\frac{1}{a}+\frac{1}{b}+\frac{1}{c}+\frac{1}{d}}} \end{array}$	EBGM05 > 2

BPNN: Bayesian Confidence Propagation Neural Network; CI: confidence interval; EBGM: Empirical Bayesian Geometric Mean; IC: information component; PRR: proportional reporting ratio; ROR: reporting odds ratio.

To address indication confounding, we additionally performed indication-stratified disproportionality analyses for UC and MS.

### Ethics approval

This study utilized the FAERS public database, which contains deidentified and aggregated data. As such, it did not require submission to a local ethics committee or institutional review board for approval.

## Results

### Distribution of AE reports for ozanimod

In this study, the term “signal” refers to a disproportionality-based reporting signal in FAERS and does not imply a confirmed causal association between ozanimod and the reported event. The AE reports related to ozanimod from the FAERS database revealed that 67.7% of the reports were from female patients, which was higher than the 27.6% reported from male patients, whereas 4.7% of the sex information was missing. The age group of 18–64.9 years accounted for 67.5% of the reports, with the second-largest group being 65–85 years, accounting for 11.3%. In terms of reporting years, the number of reports in 2021 significantly increased, accounting for 26.8% of the total reports. In terms of report sources, the majority (48.6%) of the reports were submitted by health professionals, followed by reports from patients or their family members (36.1%), reflecting the high concern for drug safety among healthcare professionals and the patient population. Geographically, most reports (93.0%) were from the United States, with relatively few reports from Germany, Poland, Russia, and Italy. Concerning the outcome of the AEs, 1.0% (75 cases) resulted in death, 0.7% (54 cases) resulted in disability, and 7.8% led to hospitalization. Most AEs occurred within 30 days of drug initiation (8.2%). The reporting frequency of AEs gradually declined over the first 180 days of treatment but subsequently increased again, with 3.1% of AEs reported after 360 days of use (Table 3).

**Table 3:** Basic Information on AEs Related to Ozanimod

Factors	Number of events (%)
Gender	
Female	4,945 (67.7%)
Male	2,014 (27.6%)
Unknown	346 (4.7%)
Age	
< 18	50 (0.7%)
18–64.9	4,930 (67.5%)
65–85	829 (11.3%)
> 85	17 (0.2%)
Reporter	
Consumer	2,637 (36.1%)
Health professional	3,552 (48.6%)
Physician	940 (12.9%)
Pharmacist	153 (2.1%)
Reported countries	
United States	6,794 (93.0%)
Germany	185 (2.5%)
Poland	41 (0.6%)
Russia	30 (0.4%)
Italy	30 (0.4%)
Report year	
2020	214 (2.9%)
2021	1,956 (26.8%)
2022	1,724 (23.6%)
2023	1,518 (20.8%)
2024	1,542 (21.1%)
2025	351 (4.8%)
Serious outcomes	
Death	75 (1.0%)
Disability	54 (0.7%)
Hospitalization	570 (7.8%)
Life-threatening	67 (0.9%)
AE occurrence time to medication date (days)	
0–30	602 (8.2%)
31–60	179 (2.5%)
61–90	144 (2.0%)
91–120	105 (1.4%)
121–150	68 (0.9%)
151–180	68 (0.9%)
181–360	218 (3.0%)
> 360	223 (3.1%)

AE: adverse event.

### Signal mining

From the second quarter of 2020 to the first quarter of 2025, a total of 15,910 AE reports were collected, with 7,305 related to ozanimod. Through signal mining, 27 SOCs and 30 PTs were identified. Table 4 ranks the SOCs by report count, with general disorders and administration site conditions (n = 3,047, ROR 1.1, PRR 1.08, IC 0.12, EBGM 1.08) having the most reports, followed by nervous system disorders (n = 2,909, ROR 2.9, PRR 2.55, IC 1.35, EBGM 2.55) and gastrointestinal disorders (n = 1,847, ROR 1.54, PRR 1.48, IC 0.56, EBGM 1.48). Based on signal strength, nervous system disorders (n = 2,909, ROR 2.9, PRR 2.55, IC 1.35, EBGM 2.55), gastrointestinal disorders (n = 1,847, ROR 1.54, PRR 1.48, IC 0.56, EBGM 1.48), and eye disorders (n = 442, ROR 1.43, PRR 1.42, IC 0.5, EBGM 1.42) ranked in the top three. Notably, in addition to the AEs clearly mentioned in the drug label, several other frequently occurring AEs were identified, such as psychiatric disorders (n = 679, ROR 0.8, PRR 0.81, IC −0.31, EBGM 0.81), renal and urinary disorders (n = 202, ROR 0.71, PRR 0.71, IC −0.49, EBGM 0.71), and benign, malignant, and unspecified (cysts and polyps) neoplasms (n = 177, ROR 0.29, PRR 0.3, IC −1.75, EBGM 0.3), which require clinical attention. Reports on hepatobiliary disorders (n = 52), endocrine disorders (n = 14), and congenital, familial, and genetic disorders (n = 4) were less frequent, and their signal strength was much lower than that of other systems, suggesting a lower reporting disproportionality for these SOCs. In summary, the AEs related to ozanimod were mainly distributed in nervous system disorders but also impact other systems and organs to some extent.

**Table 4:** Disproportionality Analysis of Ozanimod-Related AE Reports at the SOC Level

System organ class	Case reports	ROR (95% CI)	PRR (χ^2^)	EBGM (EBGM05)	IC (IC025)
General disorders and administration site conditions	3,047	1.1 (1.06–1.15)	1.08 (24.49)	1.08 (1.04)	0.12 (0.06)
Nervous system disorders	2,909	2.9 (2.78–3.02)	2.55 (2,947.53)	2.55 (2.45)	1.35 (1.29)
Gastrointestinal disorders	1,847	1.54 (1.47–1.62)	1.48 (311.11)	1.48 (1.41)	0.56 (0.49)
Investigations	1,084	1.19 (1.12–1.27)	1.18 (30.95)	1.18 (1.11)	0.24 (0.15)
Musculoskeletal and connective tissue disorders	1,045	1.32 (1.24–1.4)	1.3 (74.89)	1.3 (1.22)	1.3 (1.22)
Injury, poisoning, and procedural complications	1,009	0.47 (0.44–0.5)	0.5 (577.24)	0.5 (0.47)	−1 (−1.09)
Infections and infestations	908	1 (0.94–1.07)	1 (0.01)	1 (0.94)	0.01 (−0.09)
Psychiatric disorders	679	0.8 (0.74–0.86)	0.81 (33.06)	0.81 (0.75)	−0.31 (−0.42)
Respiratory, thoracic, and mediastinal disorders	515	0.71 (0.65–0.77)	0.72 (61)	0.72 (0.66)	−0.48 (−0.61)
Skin and subcutaneous tissue disorders	495	0.55 (0.5–0.6)	0.56 (175.44)	0.57 (0.52)	−0.82 (−0.95)
Eye disorders	442	1.43 (1.3–1.57)	1.42 (55.76)	1.42 (1.29)	0.5 (0.36)
Vascular disorders	363	1.25 (1.13–1.39)	1.24 (17.8)	1.24 (1.12)	0.32 (0.16)
Cardiac disorders	242	0.78 (0.69–0.89)	0.79 (14.5)	0.79 (0.69)	−0.35 (−0.53)
Renal and urinary disorders	202	0.71 (0.62–0.81)	0.71 (24.26)	0.71 (0.62)	−0.49 (−0.69)
Blood and lymphatic system disorders	178	0.66 (0.57–0.76)	0.66 (31.88)	0.66 (0.57)	−0.6 (−0.82)
Neoplasms benign, malignant, and unspecified (cysts and polyps)	177	0.29 (0.25–0.33)	0.3 (306.53)	0.3 (0.26)	−1.75 (−1.96)
Metabolism and nutrition disorders	160	0.52 (0.44–0.61)	0.52 (70.41)	0.52 (0.45)	−0.93 (−1.16)
Immune system disorders	119	0.65 (0.54–0.78)	0.65 (22.71)	0.65 (0.54)	−0.62 (−0.88)
Surgical and medical procedures	110	0.45 (0.38–0.55)	0.46 (72.23)	0.46 (0.38)	−1.13 (−1.4)
Reproductive system and breast disorders	72	0.74 (0.59–0.94)	0.74 (6.35)	0.74 (0.59)	−0.43 (−0.76)
Ear and labyrinth disorders	66	1.02 (0.8–1.3)	1.02 (0.04)	1.02 (0.8)	0.03 (−0.32)
Pregnancy, puerperium, and perinatal conditions	63	1.19 (0.93–1.53)	1.19 (1.99)	1.19 (0.93)	0.26 (−0.11)
Social circumstances	60	0.77 (0.6–1)	0.77 (4)	0.77 (0.6)	−0.37 (−0.74)
Hepatobiliary disorders	52	0.39 (0.29–0.51)	0.39 (50.64)	0.39 (0.3)	−1.37 (−1.75)
Product issues	28	0.09 (0.06–0.13)	0.09 (259.85)	0.09 (0.06)	−3.46 (−3.95)
Endocrine disorders	14	0.32 (0.19–0.55)	0.32 (19.8)	0.32 (0.19)	−1.63 (−2.3)
Congenital, familial, and genetic disorders	4	0.11 (0.04–0.28)	0.11 (30.43)	0.11 (0.04)	−3.24 (−4.25)

A positive signal was defined as meeting the predefined criteria of report count ≥ 3, lower limit of ROR 95% CI > 1, lower limit of PRR 95% CI > 1, EBGM05 > 2, and IC025 > 0. AE: adverse event; CI: confidence interval; EBGM: empirical Bayesian geometric mean; IC: information component; PRR: proportional reporting ratio; PT: Preferred Term; ROR: reporting odds ratio; SOC: System Organ Class.

To improve readability and facilitate comparison of the most commonly reported events, the PT-level AEs associated with ozanimod were summarized in descending order of report frequency in Table 5. The most prominent AEs included fatigue (n = 660, ROR = 3.38, PRR = 3.28, EBGM = 3.28, IC = 1.71), MS relapse (n = 548, ROR = 42, PRR = 40.59, EBGM = 39.53, IC = 5.3), headache (n = 475, ROR = 3.42, PRR = 3.34, EBGM = 3.34, IC = 1.74), dizziness (n = 314, ROR = 2.95, PRR = 2.91, EBGM = 2.91, IC = 1.54), and UC (n = 214, ROR = 14.07, PRR = 13.9, EBGM = 13.78, IC = 3.78). Notably, MS relapse and UC are not typical adverse drug reactions, but may reflect suboptimal therapeutic response, underlying disease activity, or disease progression in certain patient subpopulations. In contrast, fatigue and dizziness are nonspecific symptoms that may occur in various clinical contexts and should therefore be interpreted cautiously. Although neurological AEs have been reported with S1P receptor modulators, the FAERS database does not provide sufficient clinical detail to determine whether these nonspecific symptoms represent a defined neurological syndrome. Further clinical evaluation and case-level assessment are required to clarify their potential relevance. Other significant events included back pain (n = 201, ROR = 3.81, PRR = 3.78, EBGM = 3.77, IC = 1.91), hypertension (n = 125, ROR = 2.45, PRR = 2.44, EBGM = 2.44, IC = 1.29), urinary tract infection (n = 110, ROR = 2.53, PRR = 2.51, EBGM = 2.51, IC = 1.33), increased blood pressure (n = 102, ROR = 2.60, PRR = 2.59, EBGM = 2.59, IC = 1.37), decreased lymphocyte count (n = 99, ROR = 18.74, PRR = 18.63, EBGM = 18.41, IC = 4.20), memory impairment (n = 95, ROR = 2.83, PRR = 2.82, EBGM = 2.82, IC = 1.49), and decreased heart rate (n = 80, ROR = 7.70, PRR = 7.66, EBGM = 7.63, IC = 2.93), which are generally consistent with clinical observations and drug labeling. It should be noted that some PTs in FAERS may represent clinically related or partially overlapping concepts. For example, hypertension generally refers to a diagnosed clinical condition, whereas increased blood pressure more commonly reflects an observed increase in blood pressure as a reported finding or laboratory/vital-sign abnormality. Therefore, these two PTs should be interpreted as related cardiovascular signals rather than completely independent AEs. After excluding AEs unrelated to the drug, such as those related to surgery, medical procedures, and social circumstances, as well as events associated with FDA-approved indications or underlying disease progression, this study identified several potentially clinically meaningful AE signals, including hypoesthesia (n = 143, ROR = 4.37, PRR = 4.34, EBGM = 4.33, IC = 2.12), depression (n = 117, ROR = 2.65, PRR = 2.64, EBGM = 2.64, IC = 1.40), gait disturbance (n = 109, ROR = 2.49, PRR = 2.48, EBGM = 2.47, IC = 1.31), paresthesia (n = 104, ROR = 3.12, PRR = 3.11, EBGM = 3.10, IC = 1.63), balance disorder (n = 100, ROR = 5.31, PRR = 5.29, EBGM = 5.27, IC = 2.40), migraine (n = 89, ROR = 3.59, PRR = 3.58, EBGM = 3.57, IC = 1.84), and hemorrhage (n = 78, ROR = 3.28, PRR = 3.27, EBGM = 3.26, IC = 1.71). Similarly, gait disturbance and balance disorder are neurologically related PTs but are not identical: gait disturbance describes abnormal walking or locomotor performance, whereas balance disorder refers more broadly to impaired postural stability or equilibrium, which may or may not manifest as abnormal gait. These overlapping neurological signals should therefore be interpreted as a signal cluster suggestive of potential sensory, motor, or balance-related AEs, rather than as fully independent clinical entities. Further clinical validation and case-level assessment are needed to clarify their causal relationship with ozanimod. It should also be noted that the PT “migraine” in FAERS does not distinguish between a new diagnosis of migraine and a migraine attack, recurrence, or worsening in patients with a prior history of migraine. Therefore, this signal should be interpreted as a reported migraine-related event rather than definite new-onset migraine.

**Table 5:** Top PT-Level AE Reports Involving Ozanimod Ranked by Report Frequency

SOC	PTs	Case reports	ROR (95% CI)	PRR (χ^2^)	EBGM (EBGM05)	IC (IC025)
General disorders and administration site conditions	Fatigue	660	3.38 (3.13–3.65)	3.28 (1,057.57)	3.28 (3.03)	1.71 (1.59)
Nervous system disorders	Multiple sclerosis relapse	548	42 (38.53–45.79)	40.59 (20,611.49)	39.53 (36.26)	5.3 (5.08)
Nervous system disorders	Headache	475	3.42 (3.12–3.74)	3.34 (786.01)	3.34 (3.05)	1.74 (1.6)
Nervous system disorders	Dizziness	314	2.95 (2.64–3.3)	2.91 (395.93)	2.91 (2.6)	1.54 (1.37)
Gastrointestinal disorders	Colitis ulcerative	214	14.07 (12.29–16.12)	13.9 (2,539.94)	13.78 (12.03)	3.78 (3.5)
Musculoskeletal and connective tissue disorders	Back pain	201	3.81 (3.32–4.38)	3.78 (410.94)	3.77 (3.28)	1.91 (1.69)
General disorders and administration site conditions	Adverse event	169	9.02 (7.75–10.5)	8.93 (1,184.66)	8.88 (7.63)	3.15 (2.86)
Nervous system disorders	Multiple sclerosis	155	13.27 (11.32–15.55)	13.15 (1,725.96)	13.04 (11.13)	3.71 (3.36)
Nervous system disorders	Hypoesthesia	143	4.37 (3.71–5.16)	4.34 (367.82)	4.33 (3.68)	2.12 (1.84)
Vascular disorders	Hypertension	125	2.45 (2.06–2.93)	2.44 (106.59)	2.44 (2.05)	1.29 (1.01)
Psychiatric disorders	Depression	117	2.65 (2.21–3.18)	2.64 (119.54)	2.64 (2.2)	1.4 (1.11)
Infections and infestations	Urinary tract infection	110	2.53 (2.09–3.05)	2.51 (100.45)	2.51 (2.08)	1.33 (1.03)
General disorders and administration site conditions	Gait disturbance	109	2.49 (2.06–3)	2.48 (96.03)	2.47 (2.05)	1.31 (1.01)
Nervous system disorders	Paresthesia	104	3.12 (2.57–3.78)	3.11 (148.5)	3.1 (2.56)	1.63 (1.32)
Investigations	Increased blood pressure	102	2.6 (2.14–3.16)	2.59 (99.77)	2.59 (2.13)	1.37 (1.06)
Nervous system disorders	Balance disorder	100	5.31 (4.36–6.47)	5.29 (346.68)	5.27 (4.33)	2.4 (2.05)
Investigations	Decreased lymphocyte count	99	18.74 (15.36–22.87)	18.63 (1,631.97)	18.41 (15.09)	4.2 (3.68)
Musculoskeletal and connective tissue disorders	Muscle spasms	98	2.57 (2.1–3.13)	2.56 (92.98)	2.55 (2.09)	1.35 (1.04)
Investigations	Decreased white blood cell count	96	3.23 (2.64–3.95)	3.22 (146.73)	3.21 (2.63)	1.68 (1.36)
Nervous system disorders	Memory impairment	95	2.83 (2.31–3.47)	2.82 (111.75)	2.82 (2.3)	1.49 (1.17)
Eye disorders	Visual impairment	93	2.8 (2.28–3.43)	2.79 (106.8)	2.79 (2.27)	1.48 (1.15)
Nervous system disorders	Migraine	89	3.59 (2.92–4.42)	3.58 (165.11)	3.57 (2.9)	1.84 (1.49)
Eye disorders	Vision blurred	88	3 (2.43–3.7)	2.99 (116.31)	2.98 (2.42)	1.58 (1.24)
Injury, poisoning, and procedural complications	Maternal exposure during pregnancy	86	2.85 (2.31–3.53)	2.84 (102.85)	2.84 (2.3)	1.51 (1.17)
Investigations	Decreased heart rate	80	7.7 (6.17–9.59)	7.66 (461.3)	7.63 (6.12)	2.93 (2.5)
Blood and lymphatic system disorders	Lymphopenia	78	19.57 (15.64–24.48)	19.47 (1,349.54)	19.23 (15.37)	4.27 (3.64)
Vascular disorders	Hemorrhage	78	3.28 (2.62–4.09)	3.27 (122.5)	3.26 (2.61)	1.71 (1.34)
Cardiac disorders	Palpitations	77	3.12 (2.49–3.9)	3.11 (110.05)	3.1 (2.48)	1.63 (1.27)
Psychiatric disorders	Stress	76	3.92 (3.13–4.91)	3.9 (163.88)	3.9 (3.11)	1.96 (1.58)
Gastrointestinal disorders	Hematochezia	70	4.4 (3.47–5.56)	4.38 (182.26)	4.37 (3.45)	2.13 (1.72)

A positive signal was defined as meeting the predefined criteria of report count ≥ 3, lower limit of ROR 95% CI > 1, lower limit of PRR 95% CI > 1, EBGM05 > 2, and IC025 > 0. AE: adverse event; CI: confidence interval; EBGM: empirical Bayesian geometric mean; IC: information component; PRR: proportional reporting ratio; PT: Preferred Term; ROR: reporting odds ratio; SOC: System Organ Class.

On the basis of the data in Table 6, relapse of the underlying diseases, such as MS relapse (n = 548, ROR = 42.00, PRR = 40.59, EBGM = 39.53, IC = 5.30), progressive MS (n = 8, ROR = 40.36, PRR = 40.34, EBGM = 39.29, IC = 5.30), and proctitis ulcerative (n = 4, ROR = 32.12, PRR = 32.11, EBGM = 31.45, IC = 4.98), was particularly prominent among the main AE signals associated with ozanimod, which may reflect a suboptimal therapeutic response or natural disease progression rather than true adverse drug reactions. Uterine disorder (n = 9, ROR = 23.59, PRR = 23.57, EBGM = 23.22, IC = 4.54) also showed high signal strength, despite not being listed in the current label for ozanimod. In addition to these primary signals, less common AEs, such as traumatic fracture (n = 4, ROR = 18.65, PRR = 18.64, EBGM = 18.42, IC = 4.20), gastroenteritis *Escherichia coli* (n = 3, ROR = 17.95, PRR = 17.94, EBGM = 17.74, IC = 4.15), penile swelling (n = 3, ROR = 11.73, PRR = 11.72, EBGM = 11.64, IC = 3.54), optic neuritis (n = 3, ROR = 11.73, PRR = 11.72, EBGM = 11.64, IC = 3.54), and bladder disorder (n = 20, ROR = 8.78, PRR = 8.77, EBGM = 8.72, IC = 3.12) also presented high signal indicators, suggesting that these less frequent AEs, while rare, are of clinical significance and warrant attention in clinical practice. Enhanced pharmacovigilance monitoring is needed for these AEs.

**Table 6:** Top PT-Level Disproportionality Signals Involving Ozanimod Ranked by EBGM

SOC	PTs	Case reports	ROR (95% CI)	PRR (χ^2^)	EBGM (EBGM05)	IC (IC025)
Nervous system disorders	Multiple sclerosis relapse	548	42 (38.53–45.79)	40.59 (20,611.49)	39.53 (36.26)	5.3 (5.08)
Nervous system disorders	Progressive multiple sclerosis	8	40.36 (19.99–81.47)	40.34 (298.73)	39.29 (19.46)	5.3 (1.93)
Gastrointestinal disorders	Proctitis ulcerative	4	32.12 (11.93–86.51)	32.11 (118.02)	31.45 (11.68)	4.98 (0.84)
Investigations	Lymphocyte count abnormal	9	26.14 (13.52–50.53)	26.12 (213.66)	25.68 (13.28)	4.68 (1.97)
Investigations	Decreased T-lymphocyte count	5	26.02 (10.74–63)	26.01 (118.16)	25.58 (10.56)	4.68 (1.14)
Reproductive system and breast disorders	Uterine disorder	9	23.59 (12.21–45.58)	23.57 (191.49)	23.22 (12.02)	4.54 (1.93)
Investigations	Head magnetic resonance imaging abnormal	10	22.26 (11.92–41.58)	22.25 (199.93)	21.93 (11.74)	4.46 (2.04)
Eye disorders	Macular edema	23	19.96 (13.22–30.12)	19.93 (408.03)	19.68 (13.04)	4.3 (2.87)
Blood and lymphatic system disorders	Lymphopenia	78	19.57 (15.64–24.48)	19.47 (1,349.54)	19.23 (15.37)	4.27 (3.64)
Injury, poisoning, and procedural complications	Traumatic fracture	4	18.65 (6.95–49.99)	18.64 (65.95)	18.42 (6.87)	4.2 (0.74)
Investigations	Decreased lymphocyte count	99	18.74 (15.36–22.87)	18.63 (1,631.97)	18.41 (15.09)	4.2 (3.68)
Infections and infestations	Gastroenteritis *Escherichia coli*	3	17.95 (5.75–56.03)	17.94 (47.42)	17.74 (5.68)	4.15 (0.32)
Gastrointestinal disorders	Colitis ulcerative	214	14.07 (12.29–16.12)	13.9 (2,539.94)	13.78 (12.03)	3.78 (3.5)
Nervous system disorders	Multiple sclerosis	155	13.27 (11.32–15.55)	13.15 (1,725.96)	13.04 (11.13)	3.71 (3.36)
Reproductive system and breast disorders	Penile swelling	3	11.73 (3.76–36.53)	11.72 (29.2)	11.64 (3.74)	3.54 (0.22)
Nervous system disorders	Optic neuritis	20	11.46 (7.38–17.79)	11.44 (189.16)	11.36 (7.32)	3.51 (2.29)
Nervous system disorders	Head titubation	3	10.41 (3.34–32.39)	10.4 (25.32)	10.34 (3.32)	3.37 (0.18)
Investigations	Decreased CD4 lymphocytes	5	10.35 (4.29–24.94)	10.35 (41.92)	10.28 (4.27)	3.36 (0.83)
Nervous system disorders	Peroneal nerve palsy	11	9.67 (5.35–17.5)	9.67 (84.92)	9.61 (5.31)	3.26 (1.65)
Nervous system disorders	Central nervous system lesion	22	9.01 (5.92–13.7)	9 (155.48)	8.95 (5.88)	3.16 (2.13)
Cardiac disorders	Cardiac flutter	13	8.97 (5.2–15.48)	8.97 (91.46)	8.92 (5.17)	3.16 (1.74)
General disorders and administration site conditions	Adverse event	169	9.02 (7.75–10.5)	8.93 (1,184.66)	8.88 (7.63)	3.15 (2.86)
Renal and urinary disorders	Bladder disorder	20	8.78 (5.66–13.63)	8.77 (136.89)	8.72 (5.62)	3.12 (2.04)
Nervous system disorders	Trigeminal neuralgia	8	8.75 (4.37–17.54)	8.75 (54.58)	8.7 (4.34)	3.12 (1.26)
Eye disorders	Retinal edema	4	8.57 (3.21–22.89)	8.56 (26.57)	8.52 (3.19)	3.09 (0.47)
Ear and labyrinth disorders	Auditory disorder	4	8.12 (3.04–21.69)	8.12 (24.82)	8.08 (3.02)	3.01 (0.45)
Psychiatric disorders	Feeling of despair	9	8.06 (4.19–15.52)	8.06 (55.34)	8.02 (4.16)	3 (1.32)
Neoplasms benign, malignant, and unspecified (cysts and polyps)	Papillary thyroid cancer	3	7.78 (2.5–24.19)	7.78 (17.62)	7.74 (2.49)	2.95 (0.08)
Psychiatric disorders	Decreased interest	8	7.78 (3.88–15.6)	7.78 (47.03)	7.74 (3.87)	2.95 (1.18)
Investigations	Decreased heart rate	80	7.7 (6.17–9.59)	7.66 (461.3)	7.63 (6.12)	2.93 (2.5)

A positive signal was defined as meeting the predefined criteria of report count ≥ 3, lower limit of ROR 95% CI > 1, lower limit of PRR 95% CI > 1, EBGM05 > 2, and IC025 > 0. AE: adverse event; CI: confidence interval; EBGM: empirical Bayesian geometric mean; IC: information component; PRR: proportional reporting ratio; PT: Preferred Term; ROR: reporting odds ratio; SOC: System Organ Class.

### Indication-stratified signal comparison: UC vs. MS

To evaluate indication-specific safety, the analysis was repeated with “illnesses” filters. The stratified cohorts included 1,754 UC reports and 4,301 MS reports, with 50 overlapping reports; 1,244 reports were outside either stratum definition. Table 7 summarizes key AEs with reviewer-requested endpoints.

**Table 7:** Indication-Stratified Counts of Reviewer-Prioritized Events

AEs	UC (no. of events, no. of reports)	MS (no. of events, no. of reports)	Interpretation
Optic neuritis	0 (0)	19 (19)	Observed only in MS stratum
Gastroenteritis	0 (0)	0 (0)	No cases in both strata
Any fracture	8 (9)	21 (27)	More frequent in MS stratum
Any bladder disorder	3 (3)	27 (27)	Observed in both strata
Traumatic fracture	0 (0)	4 (4)	Observed only in MS stratum

AE: adverse event; MS: multiple sclerosis; UC: ulcerative colitis.

## Discussion

As the first oral small-molecule S1P receptor modulator approved in multiple countries for the treatment of moderately to severely active UC and relapsing forms of MS, ozanimod has demonstrated promising therapeutic effects [12]. Although the precise mechanism by which ozanimod ameliorates colonic inflammation in UC remains unclear, it is believed to exert its action primarily through high-affinity binding to S1P subtypes 1 and 5 (S1P1 and S1P5), leading to the internalization of S1P1 receptors in lymphocytes and the prevention of lymphocyte mobilization to inflammatory sites, thereby improving colonic inflammation in UC patients [11]. Phase I, II, and III clinical trials have confirmed the efficacy and favorable safety profile of ozanimod in adult patients with moderately to severely active UC [28]. However, given the occurrence of associated AEs during clinical use and the inherent limitations of clinical trial data, further studies are needed to comprehensively evaluate the long-term efficacy and safety of ozanimod in real-world applications. This study provides an in-depth analysis of the AEs associated with ozanimod in real-world settings using data from the FAERS database. The findings are discussed below.

### Distribution of AE reports and clinical significance

This study described the distribution characteristics of AE reports involving ozanimod based on data from the FAERS database. Female patients accounted for a larger proportion of reports than male patients did (67.7% vs. 27.6%). However, because FAERS does not provide reliable denominator information, such as the total number of ozanimod-exposed patients by sex, this finding should be interpreted as a reporting pattern rather than evidence of sex-based differences in drug response or the reporting frequency of AEs. Similarly, most reports involved patients aged 18–64 years (67.5%), which may reflect the age distribution of ozanimod-treated populations, reporting behavior, or the approved indications of ozanimod, rather than an age-specific risk. Geographically, most reports were submitted from the United States (93.0%). This imbalance may be related to differences in approval timing, market availability, drug utilization, pharmacovigilance systems, and reporting practices across regions. Because FAERS lacks country-specific exposure data, these results cannot be used to compare the reporting frequency of AEs between the United States and other countries. In terms of reported outcomes, hospitalization was recorded in 7.8% of reports, while death and disability were recorded in 1.0% and 0.7% of reports, respectively. These outcomes indicate that serious events were present among the submitted reports, but they do not establish that ozanimod caused these outcomes.

For reports with available onset information, the largest proportion occurred within 0–30 days after medication initiation (8.2%), while additional reports were observed after longer treatment durations, including beyond 180 days. Because onset time was missing for a substantial proportion of reports, these findings should be interpreted cautiously and should not be considered evidence of a definitive temporal risk pattern. Nevertheless, the distribution of reported onset times suggests that AE monitoring may be relevant both during treatment initiation and during longer-term therapy. Reporter information showed that healthcare professionals accounted for the largest proportion of reports (48.6%), followed by consumers (36.1%). This pattern suggests that both healthcare professionals and patients contribute substantially to FAERS reporting. Overall, these descriptive findings provide context for interpreting ozanimod-related reporting patterns but should not be interpreted as estimates of incidence, absolute risk, or causal association. The indication-stratified analyses (UC vs. MS) showed differential AE distributions. Optic neuritis and traumatic fracture events were observed only in the MS stratum, while gastroenteritis was not observed in either stratum. Fracture- and bladder-related events were present in both strata with higher burden in MS. This supports interpretation of indication-specific safety patterns, rather than attributing all reported ocular/musculoskeletal signals uniformly to UC-treated patients.

### Known AEs

Currently, evidence regarding the long-term safety and efficacy of repeated or prolonged ozanimod treatment remains relatively limited. In three pivotal clinical trials of ozanimod, including two phase 3 trials in relapsing MS (SUNBEAM and RADIANCE) and one phase 3 trial in UC (True North), approximately 3,670 patients were included overall. Across these studies, 783 patients reported AEs; however, this proportion should be interpreted cautiously because the trials involved different disease populations, treatment durations, comparator groups, and AE definitions. The most common AEs included upper respiratory infection, increased liver tests, orthostatic hypotension, urinary tract infection, back pain, hypertension, headache, pyrexia, nausea, bradycardia, and arthralgia [28–30]. This study revealed that general disorders and administration site conditions, nervous system disorders, and gastrointestinal disorders were the most commonly reported AEs, which is generally consistent with drug labeling. Clinicians should prioritize monitoring these events when using ozanimod. Notably, although “MS relapse” and “proctitis ulcerative” were high-frequency and EBGM-abnormal PTs in the AE reports, these categories are more likely to reflect disease progression or suboptimal therapeutic response rather than traditional “toxic” AEs. In addition, high-signal AEs such as macular edema and lymphocyte count abnormality are also mentioned in drug labeling, highlighting the importance of monitoring visual function and lymphocyte levels during treatment. Another notable high-signal AE, head magnetic resonance imaging abnormality, is a nonspecific imaging-related term and cannot be directly attributed to a specific neurological syndrome based on FAERS data alone. However, given that serious neurological complications, including progressive multifocal leukoencephalopathy (PML) and posterior reversible encephalopathy syndrome (PRES), are described in the labeling, abnormal neuroimaging findings should be interpreted cautiously and may warrant further clinical evaluation when accompanied by compatible neurological symptoms. Other common AEs include decreased heart rate and hypertension. These relatively mild events, however, may impact patient quality of life, warranting attention and possible adjustment of treatment on the basis of patient tolerance. In summary, the results of this study are largely consistent with existing drug safety data and further quantify adverse reaction signal strength, providing support for clinical drug monitoring.

### Potential previously unrecognized reporting signals

This study identified several potential reporting signals that were not explicitly listed in the current product labeling for ozanimod. These findings should not be interpreted as newly confirmed adverse drug reactions, but rather as hypothesis-generating pharmacovigilance signals that require further validation through case-level review and population-based studies.

#### Neuropsychiatric and emotional disturbances

Several neuropsychiatric and emotional disturbance-related reporting signals were observed, including depression, feeling of despair, and decreased interest. Among these, depression had the highest reporting frequency and signal strength. However, interpretation of these findings requires caution. Depression and anxiety are common comorbidities in patients with MS and UC, and FAERS does not provide sufficient clinical information to distinguish drug-related events from underlying disease burden, pre-existing psychiatric conditions, psychosocial stress, concomitant medications, or reporting bias [31, 32]. Therefore, these findings should be regarded as potential neuropsychiatric reporting signals rather than confirmed ozanimod-induced psychiatric AEs. Nevertheless, given the clinical burden of mood symptoms in patients with MS and UC, mental health monitoring may be considered during treatment, particularly in patients with pre-existing psychiatric symptoms.

#### Sensory and motor dysfunction

This study also observed several sensory and motor dysfunction-related reporting signals, including dizziness, hypoesthesia, paresthesia, gait disturbance, balance disorder, peroneal nerve palsy, head titubation, and head magnetic resonance imaging abnormality. Some of these PTs may overlap clinically with manifestations of underlying MS, disease relapse, demyelinating conditions, or serious neurological complications such as PML or PRES. However, these symptoms and findings are nonspecific, and FAERS lacks key clinical details, including neurological examination findings, imaging characteristics, cerebrospinal fluid results, JC virus status, disease activity, latency, dechallenge/rechallenge information, and final clinical diagnosis. Therefore, these neurological signals cannot be attributed directly to ozanimod based on the present analysis. Previous studies have suggested that S1P/S1PR signaling may participate in neuroinflammatory and remyelination-related processes [33]. However, the current FAERS analysis was not designed to evaluate these mechanisms. Accordingly, the neurological signals observed in this study should be interpreted as hypothesis-generating findings rather than evidence of direct neurotoxicity or a confirmed causal association. Clinicians should remain attentive to new or worsening neurological symptoms during treatment, especially in patients with underlying MS, and further clinical assessment is warranted when these symptoms occur.

#### Urogenital and endocrine events

In this study, several urogenital and endocrine-related reporting signals were observed, including uterine disorder, penile swelling, bladder disorder, and papillary thyroid cancer. These events are not explicitly listed in the current product labeling for ozanimod. However, these findings should be interpreted with substantial caution because some of these PT-level signals were based on very small numbers of reports. For example, penile swelling and papillary thyroid cancer had limited case counts, and FAERS does not provide sufficient clinical detail to assess latency, dose-response relationships, dechallenge or rechallenge information, prior medical history, cancer screening history, concomitant medications, or alternative explanations. Although S1P/S1PR signaling has been implicated in vascular endothelial function, immune regulation, and tumor-related biological processes, the present FAERS analysis cannot determine whether the observed urogenital or endocrine-related signals are causally related to ozanimod. In particular, the papillary thyroid cancer signal should be regarded as a hypothesis-generating observation rather than evidence that ozanimod increases the reporting signal of thyroid malignancy. Similarly, penile swelling, uterine disorder, and bladder disorder may reflect heterogeneous clinical conditions, reporting artifacts, local disease processes, infections, or comorbid conditions rather than drug-specific effects. Further evaluation using case-level clinical review, latency analysis, and population-based studies with appropriate denominator data is needed to clarify the clinical relevance of these signals.

### Recommendations for ozanimod safety monitoring

Despite ozanimod showing good efficacy in clinical trials and the overall safety being consistent with prior data, this study, which was based on real-world database mining, identified several known and potential new AE signals. This highlights the need for enhanced safety monitoring in clinical applications.

First, for known AEs, clinicians should pay particular attention to gastrointestinal, systemic, and neurological reactions—especially common symptoms such as headache, dizziness, back pain, hypertension, bradycardia, and urinary tract infections. While most of these events are relatively mild, some may impact patients’ quality of life and warrant individualized dose adjustments on the basis of tolerability. Additionally, high-signal AEs such as macular edema and lymphocyte abnormalities, although listed in the product labeling, underscore the need for routine monitoring of visual function and lymphocyte counts to ensure manageable risk.

For the potential novel AEs identified in this study, enhanced surveillance of patients’ psychological status, central nervous system function, ophthalmologic health, and urogenital symptoms is recommended. In particular, patients with preexisting psychological disorders should undergo regular mental health assessments to support their emotional well-being during treatment. Given the identification of eye-related events such as optic neuritis and retinal edema, baseline and follow-up fundus examinations are advised to enable early detection and intervention. Central nervous system-related symptoms suggestive of demyelination should prompt timely neuroimaging to evaluate underlying neurological injury.

Finally, to promote the safe and effective use of ozanimod, a comprehensive pharmacovigilance framework should be implemented in clinical practice. This includes routine monitoring of AEs, proactive patient education, and encouraging patients to report any unexpected symptoms. Such efforts can help align treatment expectations and enhance medication adherence. For patients requiring long-term or repeated dosing, individualized risk mitigation strategies—integrating imaging, laboratory data, and behavioral assessments—should be considered. Future prospective studies and population-based analyses are needed to evaluate whether these reporting signals represent true clinical associations and to further characterize the postmarketing safety profile of ozanimod.

Although ozanimod is not an oncology drug, malignancy-related safety monitoring remains clinically relevant in the context of long-term immunomodulatory therapy. Patients with UC have an increased risk of colitis-associated colorectal cancer, particularly in the setting of long-standing disease and persistent intestinal inflammation. In addition, S1P/S1PR signaling has been implicated in immune regulation, cell migration, inflammatory responses, and tumor-associated biological processes. In the present study, tumor-related signals were observed at the SOC level, and papillary thyroid cancer appeared as a PT-level signal with high disproportionality. However, the number of reports was small, and FAERS lacks detailed information on cancer history, disease duration, concomitant therapies, latency, screening intensity, and other confounding factors. Therefore, these findings should not be interpreted as evidence that ozanimod increases cancer risk. Rather, they suggest that malignancy-related events should be included in long-term postmarketing surveillance and further evaluated using prospective cohorts or claims-based databases with appropriate denominator information. Because these recommendations are based on spontaneous reporting signals rather than confirmed causal associations, they should be interpreted as precautionary considerations for clinical monitoring rather than evidence-based requirements for all patients.

### Limitations

Several limitations of this study should be considered when interpreting the findings. Because FAERS is a spontaneous reporting database, the results reflect reporting patterns rather than incidence, prevalence, absolute risk, or causal relationships. Throughout the Discussion, we therefore interpreted the findings as pharmacovigilance reporting signals rather than confirmed adverse drug reactions. First, FAERS reports are submitted voluntarily and are subject to underreporting, stimulated reporting, duplicate reporting, reporting bias, and incomplete or inaccurate information. Second, FAERS lacks reliable denominator data, such as the total number of ozanimod-exposed patients, treatment duration, prescription volume, and country-specific exposure. Therefore, the reporting proportions observed in this study cannot be used to estimate or compare the true reporting frequency of AEs across sex, age groups, countries, or treatment periods. Third, the database does not provide sufficient case-level clinical information, including medical history, disease activity, concomitant medications, latency, dose-response relationship, dechallenge/rechallenge information, laboratory results, imaging findings, or final clinical diagnosis. As a result, it is difficult to distinguish drug-related events from underlying disease progression, treatment failure, indication-related reports, comorbidities, or concomitant therapies. In addition, disproportionality analysis identifies statistical reporting signals but cannot confirm biological plausibility or causality. Some signals in this study, particularly those based on small numbers of reports or those overlapping with the treated diseases, should therefore be interpreted as hypothesis-generating findings. Further studies using case-level clinical review, prospective cohorts, claims databases, electronic health records, and other data sources with appropriate denominator information are needed to validate these signals and clarify their clinical relevance.

### Conclusion

Ozanimod is an oral S1P receptor modulator approved for the treatment of moderately to severely active UC and relapsing forms of MS. In this FAERS-based pharmacovigilance analysis, we characterized the postmarketing reporting profile of AE reports involving ozanimod. Frequently reported events included fatigue, headache, dizziness, back pain, and other general, nervous system, gastrointestinal, and musculoskeletal events. Several labeled or clinically recognized safety concerns, including decreased lymphocyte count, decreased heart rate, hypertension, macular edema, and liver test abnormalities, were also detected as reporting signals. In addition, this study observed several hypothesis-generating reporting signals not explicitly listed in the current product labeling, including depression, decreased interest, feeling of despair, hypoesthesia, gait disturbance, balance disorder, penile swelling, uterine disorder, bladder disorder, and papillary thyroid cancer. However, these findings should not be interpreted as newly confirmed adverse drug reactions or evidence of causal associations. Because FAERS lacks denominator data and detailed case-level clinical information, these signals cannot be used to estimate incidence, absolute risk, or causality. Some reported terms may also reflect underlying disease activity, treatment failure, comorbidities, concomitant medications, or reporting bias.

Overall, this study provides preliminary real-world pharmacovigilance evidence regarding AE reporting patterns involving ozanimod. The findings may help inform postmarketing surveillance and clinical awareness, but they should be considered hypothesis-generating. Further validation using prospective studies, claims databases, electronic health records, or other well-controlled real-world datasets with appropriate denominator information is needed to clarify the clinical relevance and potential causality of these signals.

## Data Availability

The data that support the findings of this study are available from the corresponding author upon reasonable request.
